# [Gly²]-GLP-2, But Not Glucagon or [D-Ala²]-GLP-1, Controls Collagen Crosslinking in Murine Osteoblast Cultures

**DOI:** 10.3389/fendo.2021.721506

**Published:** 2021-08-04

**Authors:** Aleksandra Mieczkowska, Beatrice Bouvard, Erick Legrand, Guillaume Mabilleau

**Affiliations:** ^1^Univ Angers, GEROM, SFR ICAT, Angers, France; ^2^CHU Angers, Rheumatology Department, Angers, France; ^3^CHU Angers, Bone Pathology Unit, Angers, France

**Keywords:** GLP-2, osteoblast (OB), collagen cross-linking, bone quality, GLP-1

## Abstract

Bone tissue is organized at the molecular level to resist fracture with the minimum of bone material. This implies that several modifications of the extracellular matrix, including enzymatic collagen crosslinking, take place. We previously highlighted the role of several gut hormones in enhancing collagen maturity and bone strength. The present study investigated the effect of proglucagon-derived peptides on osteoblast-mediated collagen post-processing. Briefly, MC3T3-E1 murine osteoblasts were cultured in the presence of glucagon (GCG), [D-Ala²]-glucagon-like peptide-1 ([D-Ala²]-GLP-1), and [Gly²]-glucagon-like peptide-2 ([Gly²]-GLP-2). Gut hormone receptor expression at the mRNA and protein levels were investigated by qPCR and Western blot. Extent of collagen postprocessing was examined by Fourier transform infrared microspectroscopy. GCG and GLP-1 receptors were not evidenced in osteoblast cells at the mRNA and protein levels. However, it is not clear whether the known GLP-2 receptor is expressed. Nevertheless, administration of [Gly²]-GLP-2, but not GCG or [D-Ala²]-GLP-1, led to a dose-dependent increase in collagen maturity and an acceleration of collagen post-processing. This mechanism was dependent on adenylyl cyclase activation. In conclusion, the present study highlighted a direct effect of [Gly²]-GLP-2 to enhance collagen post-processing and crosslinking maturation in murine osteoblast cultures. Whether this effect is translatable to human osteoblasts remains to be elucidated.

## Introduction

Bone tissue is a two-component material made primarily of poorly crystalline hydroxyapatite tablets and type I collagen-based organic matrices (1). The mineral component confers stiffness to the bone tissue (2, 3) while collagen confers a degree of tensile strength, ductility, and toughness (4, 5). Type I collagen biosynthesis is a complex process and includes several intracellular post-translational modifications in osteoblast including assembly of pro-collagen chains, secretion, extracellular processing, and crosslinking to form a mature functional organic matrix (6). The resulting collagen molecules assemble into collagen type I fibrils that are subsequently stabilized as a result of oxidative deamination of ε-amino groups of specific lysine and hydroxylysine residues by lysyl oxidase, forming aldehyde moieties (7). Aldehyde moieties are extremely reactive and their interactions with other aldehydes in the side chain of amino acids present in the primary structure of type I collagen result in the formation of covalent crosslinks, critical for a functional mature collagen. The formation of collagen fibrils has been described as entropy driven, similar to the self-assembly of microtubules or actin filaments (8). However, several non-collagenic proteins found in the bone extracellular matrix have been shown to regulate collagen deposition and fibril formation. Among them, biglycan and decorin, two small proteoglycans, play a major role in collagen deposition and fibril formation as demonstrated by abnormal collagen fibrils in deficient animals (9, 10). Furthermore, functional lysyl hydroxylase and lysyl oxidase, two key enzymes involved in post-translational modifications of collagen molecules, are also compulsory for normal collagen deposition and fibril organization (11).

A growing body of evidence suggested that several peptides produced by enteroendocrine cells are key in the maintenance of bone quality (12–14). Depending on tissue-specific splicing, proglucagon mRNA gives rise to several peptide including glucagon (GCG), glicentin-related peptide, oxyntomodulin, glucacon-like peptide-1 (GLP-1), and glucagon-like peptide-2 (GLP-2) (15). Administration of exogenous oxyntomodulin, GLP-1, or GLP-2 has previously evidenced beneficial effects on bone quality and especially collagen maturity *in vivo* (16–18). However, despite the fact that GLP-1 and GLP-2 receptors have previously been evidenced in murine and human osteoblasts (19, 20), little is known whether the observed effects are due to direct activation of osteoblast cells.

The aim of the present study was to investigate whether GCG, [D-Ala²]-GLP-1, and [Gly²]-GLP-2 directly affect collagen biosynthesis and processing in osteoblast cultures.

## Materials and Methods

### Reagents

Alpha-MEM, penicillin, and streptomycin were purchased from Gibco (Wokingham, UK). Fetal bovine serum was purchased from Eurobio Scientific (Les Ulis, France). [D-Ala²]-GLP-1, GLP-1, [Gly²]-GLP-2, GLP-2, and GCG were synthesized by Genecust (Boynes, France). Purity and molecular mass of peptides were determined by reverse-phase HPLC and mass spectrometry. All other chemicals were purchased from Sigma-Aldrich (Poole, UK) when otherwise stated.

### Cell Culture

MC3T3-E1 cells are phenotypically normal osteoblasts, derived from fetal mouse calvaria, that can differentiate into bone-forming osteoblasts and the deposition of bone-like extracellular matrix (21). MC3T3-E1 cells were purchased from the American Type Culture Collection (ATCC, Teddington, UK). Cells were grown and expanded at a ratio of 1:4 in propagation medium containing alpha-MEM supplemented with 10% FBS, 100 U/ml penicillin, and 100 μg/ml streptomycin in a humidified atmosphere enriched with 5% CO_2_ at 37°C. For collagen deposition studies, cells were detached with trypsin-EDTA and plated at a density of 1.5 × 10^4^ cells/cm^2^ and grown to confluence in propagation medium. Confluence was reached within 48 h. At confluence, the propagation medium was replaced by the differentiation medium containing alpha-MEM supplemented with 10% FBS, 100 U/ml penicillin, 100 μg/ml streptomycin, 50 µg/ml ascorbic acid, and various concentrations of proglucagon-derived peptides. This day was considered as day 1. The differentiation medium was replenished every 2 days. 2′,5′-Dideoxyadenosine (DDA) was used as an adenylyl cyclase inhibitor at a concentration of 50 μM. This concentration has been used previously to dramatically reduce the level of cAMP in MC3T3-E1 cells without affecting osteoblast viability (13).

### Receptor Expression

Sequences encoding the murine *Gcgr*, *Glp1r*, and *Glp2r* were synthesized and cloned into pcDNA3.1(+) vector between *Nhe*I and *Eco*RI sites of the multiple cloning sites. The resulting vectors were sequenced for validation (GeneCust, Boynes, France). CHO-K1 cells were purchased from ATCC (Teddington, UK). Cells were grown and expanded at a ratio of 1:5 in propagation medium containing F-12 supplemented with 10% FBS 100 U/ml penicillin and 100 μg/ml streptomycin in a humidified atmosphere enriched with 5% CO_2_ at 37°C. CHO-K1 cells were transfected with plasmids encoding proglucagon-derived peptide receptors or the empty vector using Invitrogen™ Lipofectamine™ 3000 as recommended by the manufacturer. Twenty-four hours post-transfection, total RNA was extracted using Nucleozol (Macherey-Nagel GmbH, Düren, Germany) and mRNA were purified with NucleoSpin columns (Macherey-Nagel) and stored at −80°C until use. In parallel, MC3T3-E1 cells were grown in propagation medium and total RNA was isolated 48 h post-seeding as for CHO-K1 cells. In a pilot study, we did not evidence changes in expression between 24 h, 48 h, and 72 h. One microgram of total RNA was reverse transcribed using random hexamer primers and SuperScript II reverse transcriptase (Invitrogen, France). Amplification was performed in duplicate with iQ SYBR Supermix (BioRad, Courtaboeuf, France) using a Light Cycler 480 machine (Roche, Mannheim, Germany). Primer sequences have been designed by the Cell and molecular analyses platform of the University of Angers (http://sfricat.univ-angers.fr/fr/plateformes/pacem-analyse-cellulaire-et-moleculaire.html) and are provided in **Table 1**. Gene expression was calculated using the comparative Ct method with the normalization of the target to the housekeeping gene *B2m*.

**Table 1:** PCR primers used in this study.

Gene	Forward primer	Reverse primer	Gene ID
*B2m*	CTCGCTCGGTGACCC	CGGGTGGAACTGTGTTACG	NM_009735.3
*Gcgr*	CCCAGGTAATGGACTTTTTGT	GTACTTGTCGAAGGTTCTGTTAC	NM_008101.2
*Glp1r*	CATTCTCTTTGCTATCGGCG	GAGAGTCAGAGTGGACTTGG	NM_021332.2
*Glp2r*	GCAAAATCAACCCTGCTCC	CGAGCTAATAAGAAGCGGC	NM_175681.3

At the protein level, receptor expression was investigated by Western blot. Briefly, empty pcDNA3.1(+) vector or vectors containing proglucagon-derived peptide receptor sequences were transfected as above into CHO-K1 cells. Forty-eight hours post-transfection, cells were lysed with RIPA buffer (Thermo Fisher Scientific, Rockford, IL). MC3T3-E1 were grown in propagation medium and lysed with RIPA buffer as described for CHO-K1 cells. Cell lysates were spun at 13,000 *g* for 15 min at 4°C and stored at −80°C until use. Forty micrograms of protein lysates were loaded and run on a 4%–12% bis Tris plus bolt gel (Thermo Fisher Scientific) and blotted onto a PVDF membrane. The PVDF membranes were blocked with 5% bovine serum albumin (BSA) in Tris buffered saline for 1 h and membranes were incubated overnight at 4°C with specific antibodies for GCGr (Ref NLS4256, Novus Biologicals, Noyal Châtillon sur Seiche, France), GLP-1r (Ref NBP1-97308, Novus Biologicals), or GLP-2r (Ref PA5-76959, Invitrogen, Rockford, IL). Subsequently, the membranes were washed in TBS and incubated with a rabbit IgG HRP-conjugated antibody for 1 h at room temperature. Immunoreactive bands were visualized using an ECL kit (Amersham Biosciences).

### Intracellular cAMP Determination and Phospho-Proteome Array

For intracellular cAMP determination and phospho-proteome array, MC3T3-E1 cells were plated at a density of 1.5 × 10^4^ cells/cm^2^ and grown for 48 h in alpha-MEM supplemented with 5% FBS, 5% bovine calf serum, 100 U/ml penicillin, and 100 μg/ml streptomycin in a humidified atmosphere enriched with 5% CO_2_ at 37°C. After 48 h, cells were starved in alpha-MEM supplemented with 0.5% BSA for 16 h and then incubated for 15 min in alpha-MEM supplemented with 0.5% BSA and 1 mM IBMX (cAMP assay only) prior to stimulation with proglucagon-derived peptides. After 45 min, cells were washed with ice-cold PBS, incubated in RIPA buffer, and centrifuged at 13,000 *g* for 10 min. Supernatants were collected and stored at −80°C until use. Cyclic AMP determination was performed with a fluorometric commercially available kit (R&D Systems Europe, Abingdon, UK) according to the manufacturer’s recommendations. Phospho-proteome array was performed using a commercially available kit (ref ARY003C, R&D Systems Europe, Abingdon, UK) according to the manufacturer’s recommendations.

### Amine Oxidase Activity

Amine oxidase activity was assessed in the cell culture supernatant after 13 days of culture and stimulation with proglucagon-derived peptides. This period of time corresponds to the highest expression of LOX (11). Oxidase activity was determined using a fluorometric assay developed initially by Palamakumbura et al. (22). At day 12, the differentiation medium was replaced by phenol red-free DMEM containing 0.1% BSA, 50 µg/ml ascorbic acid, and proglucagon-derived peptides. After 24 h, cell culture supernatants were collected, centrifuged at 3,000 rpm for 30 min at 4°C, aliquoted, and stored at −80°C until use. Amine oxidase activity was determined in culture supernatants after incubation at 37°C for 30 min in the presence of 1.2 M urea, 50 mM sodium borate (pH 8.2), 1.3 nmol H_2_O_2_, 1 UI/ml horseradish peroxidase, 10 mM diaminopentane, and 10 µM Ampliflu™ red in opaque 96-well plates. At the end of the incubation period, the plate was placed on ice and fluorescence was read using an M2 microplate reader (Molecular devices, St Gregoire, France) with excitation and emission wavelength was set up at 563 nm and 587 nm, respectively.

### Fourier Transform Infrared Microspectroscopy (FTIRM)

For determination of enzymatic collagen crosslinking, the same plate used for LOX assay was decellularized prior to examination by FTIRM. Briefly, cells were rinsed with cold PBS and incubated in decellularization medium consisting of 0.2 M cacodylate buffer (pH 7.4), enriched with 0.1% Triton X-100, for 4 h at room temperature. Then, cultures were rinsed abundantly with milliQ water, fixed in absolute ethanol, scraped off the culture dish, and transferred onto BaF_2_ windows where it was air-dried as reported previously (13).

Collagen crosslinking was evaluated by FTIRM using a Bruker Vertex 70 spectrometer (Bruker optics, Ettlingen, Germany) interfaced with a Bruker Hyperion 3000 infrared microscope equipped with a standard single-element Mercury Cadmium Telluride (MCT) detector. Mid-infrared spectra were recorded at a resolution of 4 cm^−1^, with an average of 32 scans in transmission mode. Background spectral images were collected under identical conditions from the same BaF_2_ windows at the beginning and end of each experiment to ensure instrument stability. Water vapor was corrected prior to baseline correction. Post-processing was performed with a lab-made Matlab routine (R2020b, The Mathworks, Natick, CA) and included Mie scattering correction, second-derivative spectroscopy, and curve fitting routines. Trivalent collagen crosslink content (Area ratio 1660 cm^-1^/Amide 1), divalent collagen crosslink content (Area ratio 1690 cm^-1^/Amide 1), and collagen maturity (area ratio 1660/1690 cm^-1^) were computed as reported previously (23).

### Statistical Analysis

Each experiment has been replicated at least three times. Results were expressed as mean ± standard deviation (SD). Kruskal–Wallis test with Dunn’s multiple comparisons was used when more than two groups were present in the series. When only two groups were present in the series, non-parametrical Mann–Whitney test was used (GraphPad Prism 6.0, San Jose, CA). Differences at *p* < 0.05 were considered to be significant.

## Results

### Activation of MC3T3-E1 Osteoblasts With [Gly²]-GLP-2 But Not GCG or [D-Ala²]-GLP-1

**Figure 1:**
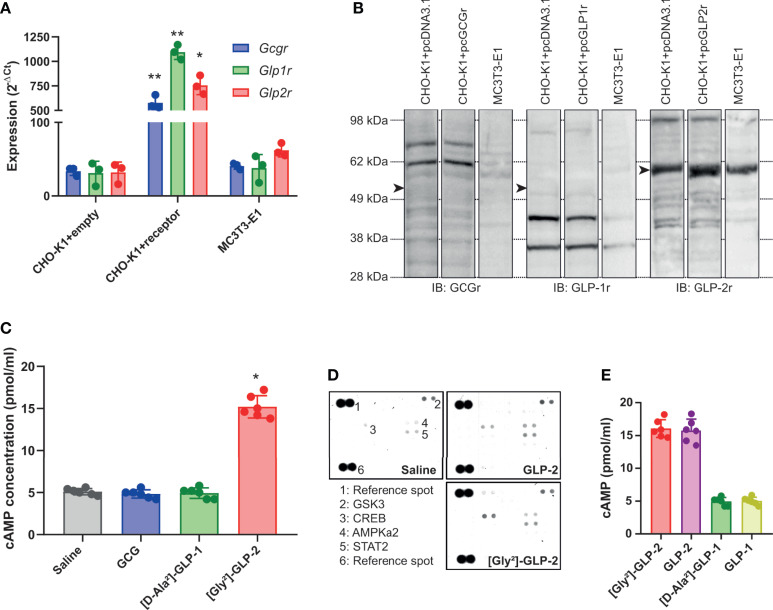
Expression and activation of proglucagon-derived peptide receptors in murine osteoblasts. **(A)** Relative expression of glucagon receptor (*Gcgr*), glucagon-like peptide-1 receptor (*Glp1r*), and glucagon-like peptide-2 receptor (*Glp2r*) was investigated by qPCR. CHO-K1 cells transfected with the empty pcDNA3.1 vector was used as negative control. CHO-K1 cells transfected with the pcDNA 3.1 vector encoding the *Gcgr*, *Glp1r*, or *Glp2r* sequence were used as positive control. Expression in MC3T3-E1 cells was used to assess expression in osteoblast cells. **(B)** Presence of GCGr, GLP-1r, and GLP-2r was determined by Western blot with commercially available antibodies. Arrowheads point out at the theoretical location of receptor. **(C)** Intracellular levels of cAMP were determined in MC3T3-E1 cells 45 min after treatment with 50 pM of GCG, [D-Ala²]-GLP-1, or [Gly²]-GLP-2. **(D)** Phospho-proteome array in MC3T3-E1 cells treated with either saline or 50 pM of GLP-2 or its long-lasting analog [Gly²]-GLP-2. **(E)** Intracellular levels of cAMP were determined in MC3T3-E1 cells 45 min after treatment with 50 pM of GLP-2 or [Gly²]-GLP-2. *p < 0.05 and **p < 0.01 vs. saline-treated cultures.

We first examined whether MC3T3-E1 osteoblast cells expressed the known proglucagon-derived peptide receptors. As a positive control, we used CHO-K1 cells transfected with plasmids encoding the murine receptors. As evidenced in **Figure 1A**, transcripts of all three receptors were present in receptor-transfected CHO-K1 cells. However, in MC3T3-E1 cells, the level of expression of *Gcgr* and *Glp1r* was not significantly different as observed in CHO-K1 cells transfected with empty vectors. However, it is worth noting that *Glp2r* expression in MC3T3-E1 cells, although not significant as compared with CHO-K1 cells transfected with the empty vector, almost reached statistical significance with a *p*-value of 0.0636. At the protein level, surprisingly, commercial antibodies sold as specific of GCGr and GLP-1r failed to evidence any sign of higher expression in receptor-transfected CHO-K1 cells as compared with empty vector-transfected cells (**Figure 1B**). A stronger band was observed in GLP-2r-transfected CHO-K1 cells as compared with empty vector-transfected cells. However, as a band was also present in empty vector-transfected CHO-K1 cells, it is difficult to ascertain whether GLP-2r is expressed by MC3T3-E1 osteoblast cells. We also performed a functional assay where MC3T3-E1 cells were stimulated with 50 pM of GCG, [D-Ala²]-GLP-1, or [Gly²]-GLP-2 (**Figure 1C**). [Gly²]-GLP-2, but not GCG or [D-Ala²]-GLP-1, significantly increased intracellular cAMP levels as compared with saline-treated cells (*p* = 0.0277), suggesting that MC3T3-E1 osteoblast cells are capable to respond directly to [Gly²]-GLP-2. In order to verify whether such response was specific to [Gly^2^]-GLP-2 or could be extrapolated to native GLP-2 peptide, we investigated the extent of activation in intracellular pathways in MC3T3-E1 cells. Interestingly, [Gly²]-GLP-2 and GLP-2 activated the same intracellular pathways represented by CREB (Ser133), AMPKa2 (Thr172), and STAT2 (Tyr689) (**Figure 1D**). Furthermore, in order to ascertain whether increase of intracellular cAMP in MC3T3-E1 cells was restricted to [Gly²]-GLP-2, we compared head-to-head the extent of cAMP elevation with unmodified peptide, and we did not evidence any significant difference (**Figure 1E**). In order to verify if the lack of cAMP response, in MC3T3-E1 cells, treated with [D-Ala²]-GLP-1 was restricted to the use of the long-lasting analog, we also compared head-to-head the extent of intracellular cAMP response with native peptide. GLP-1 and [D-Ala²]-GLP-1 did not show any sign of cAMP increase and activation.

### Effects of Proglucagon-Derived Peptides on Lysyl Oxidase Activity and Collagen Maturity

**Figure 2:**
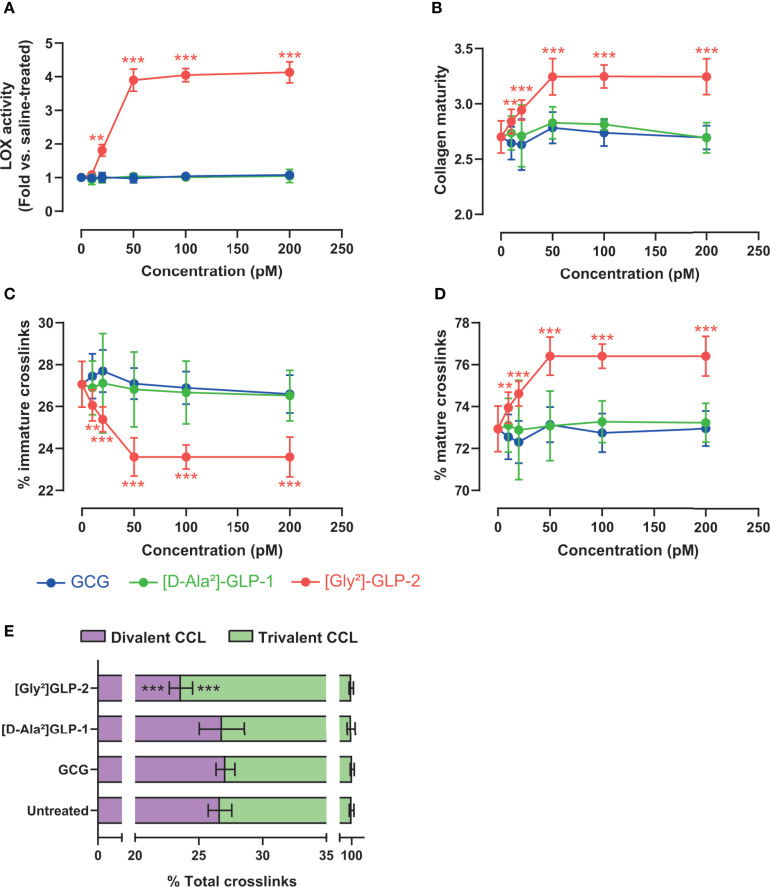
Proglucagon-derived peptides and collagen matrix quality. **(A)** Lysyl oxidase activity and **(B)** collagen maturity, expressed as the ratio of mature trivalent/immature divalent collagen crosslinks, are dose-dependently increased in the presence of [Gly²]-GLP-2, but not GCG or [D-Ala²]-GLP-1. **(C)** Percentage of immature and **(D)** mature collagen crosslinks in the extracellular matrix. **(E)** Normalized enzymatic collagen crosslinking in the extracellular matrix at 50 pM. **p < 0.01 and ***p < 0.001 *vs*. untreated cultures.

We next thought to ascertain whether proglucagon-derived peptides were capable of modulating lysyl oxidase activity, a key enzyme involved in enzymatic crosslinking of collagen fibers in bone tissue, and possibly enzymatic collagen crosslinking in MC3T3-E1 osteoblast cultures. As shown in **Figure 2A**, only [Gly²]-GLP-2 was capable of significantly enhancing lysyl oxidase activity (*p* < 0.0001), in a dose-dependent manner up to 50 pM. Neither GCG nor [D-Ala²]-GLP-1 significantly enhanced lysyl oxidase activity. Collagen maturity, representing the ratio of mature trivalent collagen crosslinks over immature divalent collagen crosslinks, was significantly dose-dependently increased with [Gly²]-GLP-2 to reach a maximum at concentrations of 50 pM and above (**Figure 2B**). However, this parameter was unchanged in the presence of increasing concentrations of GCG or [D-Ala²]-GLP-1. In order to better apprehend the impact of proglucagon-derived peptides on collagen maturity, we thought to determine changes in immature and mature collagen crosslinks. Here again, [Gly²]-GLP-2, but not GCG or [D-Ala²]-GLP-1, significantly reduced dose-dependently the extent of immature crosslinks and increased the amount of mature crosslinks, suggesting an acceleration of collagen crosslink maturation (**Figures 2C, D**). Indeed, when divalent and trivalent collagen crosslinks were plotted at the concentration of 50 pM (**Figure 2E**), it appeared obvious that conversion of immature to mature crosslink was enhanced in the presence of [Gly²]-GLP-2.

### [Gly²]-GLP-2 Enhances LOX Activity and Collagen Maturity Through a cAMP-Dependent Pathway

**Figure 3:**
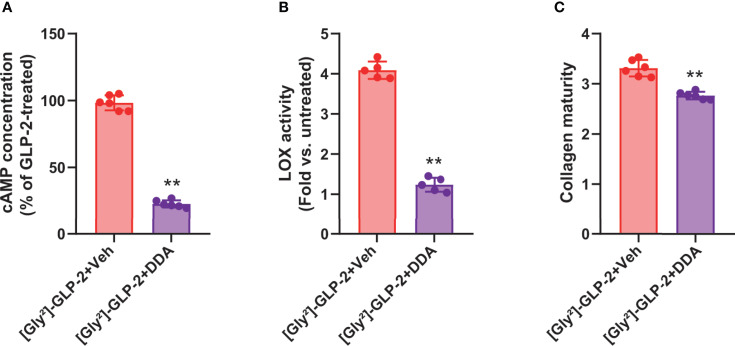
GLP-2 action on collagen maturity is dependent on adenylyl cyclase activity. MC3T3-E1 osteoblast cells were incubated with a specific adenylyl cyclase inhibitor (DDA) prior to incubation with 50 pM [Gly²]-GLP-2. **(A)** cAMP, **(B)** LOX activity, and **(C)** collagen maturity in osteoblast cultures. **p < 0.01 *vs*. vehicle-treated cultures.

As [Gly²]-GLP-2 induced a significant rise in intracellular cAMP, we thought to determine the contribution of this pathway in MC3T3-E1 osteoblast cultures. As presented in **Figure 3A**, and as expected, the use of 2′,5′-dideoxyadenosine (DDA) significantly reduced the intracellular levels of cAMP as compared with vehicle-treated cultures (*p* = 0.002). Subsequently, we also examined the impact of cAMP inhibition on LOX activity (**Figure 3B**). Interestingly, the use of DDA significantly reduced LOX activity to levels similar to saline-treated cultures (*p* = 0.008). Consequently, the collagen maturity was also altered and reduced by 16% ± 2% in [Gly²]-GLP-2+DDA-treated cultures as compared with [Gly²]-GLP-2+vehicle-treated cells (*p* = 0.002) (**Figure 3C**).

## Discussion

Several lines of evidence suggest that peptides produced by enteroendocrine cells are beneficial for bone quality and strength (16, 24–28). Indeed, GLP-2 was previously shown to reduce circulating markers of bone resorption rapidly after administration and to enhance bone mineral density in post-menopausal women (29, 30). The present study provides new insights into the effects of proglucagon-derived peptides on osteoblasts and highlights that [Gly²]-GLP-2 but not GCG or [D-Ala²]-GLP-1, directly participates in collagen post-processing through activation of adenylyl cyclase and the production of cAMP.

The present study could not demonstrate with certitude the presence of the known GLP-2 receptor at the surface of MC3T3-E1 cells. MC3T3-E1 cells are murine preosteoblasts, similar to calvarial osteoblasts, that can be differentiated into mature osteoblasts in the presence of ascorbic acid (21). Previously, it has been postulated that GLP-2 exerts its skeletal effects through binding in parathyroid glands and reduction of parathyroid hormone secretion (31, 32). Unfortunately, the specificity of the anti-GLP-2r used in this study was not enough to validate undoubtedly the presence of GLP-2r in osteoblasts. Specificity of GLP-2r antibodies is a real issue, which has also been previously reported by others (33, 34) and indeed, and up to now, we did not validate a suitable antibody. Previously, expression of the GLP-2r had been detected in human osteosarcoma-derived MG-63 and TE-85 cell lines (19). However, more recent human data, acquired in human bone marrow mesenchymal stem cell differentiated towards osteoblasts, suggest the absence of the known GLP-2r (35). Our data gathered in the MC3T3-E1 cells are in agreement with such observation, but it is worth noting that we cannot exclude a low level of expression of this GLP-2r in murine osteoblasts or alternative splicing that could hamper its detection with our primer pair as already evidenced by others recently in the testis (36). However, and more interestingly, administration of [Gly²]-GLP-2 or GLP-2 in the osteoblast cultures resulted in increased concentration of intracellular cAMP, suggesting that these cells were still capable to respond to these peptides. Further studies are required to fully elucidate the mechanism of action of GLP-2 in osteoblasts. However, it is worth noting that such observations have already been reported for GLP-1 in the past with the possible presence of a secondary receptor in MC3T3-E1 cells (37). However, whether such receptor is specific to GLP-1, or shared with other molecules, remains to be elucidated. Nevertheless, the data presented in the present study suggest that if this receptor is present in MC3T3-E1 cells, it is not involved in collagen post-processing.

Administration of [Gly²]-GLP-2 into mature murine osteoblast cultures resulted in higher lysyl oxidase activity, a faster conversion of immature to mature collagen crosslinks, and ultimately to a higher maturity of the newly deposited extracellular matrix. This process was dependent on adenylyl cyclase as the use of a specific adenylyl cyclase inhibitor, 2’,5’dideoxyadenosine, blocked this effect. This is interesting and in agreement with already published literature where higher collagen maturity was observed in GLP-2-treated ovariectomized animals (16). Previously, effects of GLP-2 action on bone resorption have been hypothesized through a parathyroid hormone-dependent mechanism (31, 32). More recent literature is in agreement with such effects (35). However, in the present study, we also evidenced that the action of [Gly²]-GLP-2 on bone physiology is due to a direct stimulation of cAMP in osteoblasts. The respective contribution of direct vs. indirect effects of GLP-2 on collagen maturity *in vivo* requires to be further elucidated.

Importantly, in this study, we failed to evidence any effects of GCG or [D-Ala²]-GLP-1 on osteoblast-mediated actions. We did not observe the expression of GCGr or GLP-1r in osteoblasts and here again, the specificity of commercial antibodies directed to GCGr or GLP-1r epitopes was not satisfactory to highlight unambiguously the presence of these receptors in these cells. Nevertheless, we previously evidenced the lack of the known *Glp1r* expression in murine bone (26) whereas several authors demonstrated its expression in bone cell and tissue (19, 20, 38). Despite no receptor expressed on osteoblast, GLP-1 administration in several preclinical models of bone disorders demonstrated a potential for enhancing collagen post-processing, suggesting that the actions of GLP-1 are extraskeletal (12, 17, 18, 39). Furthermore, in clinical studies, GLP-1 was also capable of reducing osteoclast resorption as indicated by a reduction in CTx levels but had no effects on circulating marker of bone formation (40). However, although the presence of a putative GLP-1r in osteoclast remains to be fully demonstrated, it is plausible that the effects of GLP-1 on osteoclast resorption might be indirect.

Interestingly, GLP-2 is the second gut hormone, after glucose-dependent insulinotropic polypeptide (GIP), to exhibit direct effects on osteoblast-mediated collagen post-processing. Indeed, GIP is capable of enhancing directly LOX activity and collagen crosslinking in a cAMP-dependent manner, similar to what is observed with [Gly²]-GLP-2 (13). Recently, it was evidenced that GIP exerts this effect through activation of adenylyl cyclase, protein kinase A, phosphorylation of beta-catenin at Ser675, and complexation with TCF/LEF transcription factor in the LOX promoter (41). It remains to be ascertained whether GLP-2 exerts its effect through similar intracellular signaling pathways in osteoblasts. Nevertheless, it was previously shown that GLP-2 effects on circulating markers of bone remodeling were not inhibited by GIP_3-30_, a known GIPr antagonist (32), suggesting that GIP and GLP-2 would exhibit a synergistic action. It would be interesting to evaluate whether both peptides act synergistically to improve collagen post-processing.

In conclusion, the present study highlighted a direct effect of [Gly²]-GLP-2 to enhance collagen post-processing and crosslinking maturation in murine osteoblast cultures despite the fact that the known GLP-2r was not evidenced with certainty. Whether this effect is translatable to human osteoblasts remains to be elucidated.

## Data Availability Statement

The original contributions presented in the study are included in the article/supplementary material. Further inquiries can be directed to the corresponding author.

## Author Contributions

BB, EL, and GM contributed to conception and design of the study. AM and GM performed experiments. AM and GM performed analyses. GM wrote the first draft of the manuscript. All authors contributed to the article and approved the submitted version.

## Funding

This work was supported by a grant from SATT Ouest Valorisation (Grant DV2541). 

## Conflict of Interest

AM and GM hold a patent on the dual GIP/GLP-2 analogs for the treatment of bone disorders (WO2020169792A1).

The remaining authors declare that the research was conducted in the absence of any commercial or financial relationships that could be construed as a potential conflict of interest.

## Publisher’s Note

All claims expressed in this article are solely those of the authors and do not necessarily represent those of their affiliated organizations, or those of the publisher, the editors and the reviewers. Any product that may be evaluated in this article, or claim that may be made by its manufacturer, is not guaranteed or endorsed by the publisher.
